# Individualized Dosing With High Inter-Occasion Variability Is Correctly Handled With Model-Informed Precision Dosing—Using Rifampicin as an Example

**DOI:** 10.3389/fphar.2020.00794

**Published:** 2020-05-27

**Authors:** Lina Keutzer, Ulrika S. H. Simonsson

**Affiliations:** Department of Pharmaceutical Biosciences, Uppsala University, Uppsala, Sweden

**Keywords:** model-informed precision dosing, inter-occasion variability, tuberculosis, rifampicin, Bayesian forecasting, precision dosing

## Abstract

Rifampicin exhibits complexities in its pharmacokinetics (PK), including high inter-occasion variability (IOV), which is challenging for dose individualization. Model-informed precision dosing (MIPD) can be used to optimize individual doses. In this simulation-based study we investigated the magnitude of IOV in rifampicin PK on an exposure level, the impact of not acknowledging IOV when performing MIPD, and the number of sampling occasions needed to forecast the dose. Subjects with drug-susceptible tuberculosis (TB) were simulated from a previously developed population PK model. To explore the magnitude of IOV, the area under the plasma concentration-time curve from time zero up to 24 h (AUC_0–24h_) after 35 mg/kg in the typical individual was simulated for 1,000 sampling occasions at steady-state. The impact of ignoring IOV for dose predictions was investigated by comparing the prediction error of a MIPD approach including IOV to an approach ignoring IOV. Furthermore, the number of sampling occasions needed to predict individual doses using a MIPD approach was assessed. The AUC_0–24h_ in the typical individual varied substantially between simulated sampling occasions [95% prediction interval (PI): 122.2 to 331.2 h mg/L], equivalent to an IOV in AUC_0–24h_ of 25.8%, compared to an inter-individual variability of 25.4%. The median of the individual prediction errors using a MIPD approach incorporating IOV was 0% (75% PI: −14.6% to 0.0%), and the PI for the individual prediction errors was narrower with than without IOV (median: 0%, 75% PI: −14.6% to 20.0%). The most common target dose in this population was forecasted correctly in 95% of the subjects when IOV was included in MIPD. In subjects where doses were not predicted optimally, a lower dose was predicted compared to the target, which is favorable from a safety perspective. Moreover, the imprecision (relative root mean square error) and bias in predicted doses using MIPD with IOV decreased statistically significant when a second sampling occasion was added (difference in imprecision: −9.1%, bias: −7.7%), but only marginally including a third (difference in imprecision: −0.1%, bias: −0.1%). In conclusion, a large variability in exposure of rifampicin between occasions was shown. In order to forecast the individual dose correctly, IOV must be acknowledged which can be achieved using a MIPD approach with PK information from at least two sampling occasions.

## Introduction

Individualized dosing is important to improve treatment outcomes by avoiding toxicity while still achieving optimal efficacy in each individual patient. The goal is to ensure optimal drug exposure in an individual patient, especially for drugs with a narrow therapeutic window, regimens with a risk of drug-drug interactions, patients with polypharmacy, and special patient subpopulations. Different approaches are being utilized to reach this goal, ranging from regression analysis over decision trees to model-based strategies, depending on which purpose needs to be fulfilled (Darwich et al., 2017). One of the strategies is model-informed precision dosing (MIPD). MIPD is an approach where information from a population pharmacokinetic (POPPK) or physiology-based pharmacokinetic (PBPK) model in combination with individually observed plasma drug concentrations is utilized to forecast the dose that leads to the most optimal exposure in an individual patient (Darwich et al., 2017; Keizer et al., 2018). Further, MIPD can incorporate not only pharmacokinetics (PK) but also efficacy and safety aspects in the individual dose prediction, i.e. predict the dose given not only a POPPK or PBPK model, but also given pharmacokinetic-pharmacodynamic (PKPD) models. This is superior to classical therapeutic drug monitoring (TDM), which does not predict an optimal individual dose, but only compares the individual exposure to a target and evaluates if the individual exposure is too high or too low. Traditionally, TDM is used to individualize a dose based on measured plasma drug concentrations to account for random, non-predictable differences in PK between individuals, referred to as inter-individual variability (IIV) or between-subject variability (BSV). However, a high inter-occasion variability (IOV) [*synonym*: between-occasion variability (BOV)] in PK can be problematic for classical TDM, i.e. the random variability which is variability not due to IIV, but rather variability within an individual between occasions (sampling or dosing occasions) (Karlsson and Sheiner, 1993). This is a random variability which cannot be explained by known factors such as time-varying PK (i.e. enzyme auto-induction), concomitant food intake, changes in creatinine clearance, etc. It is commonly stated that TDM should not be performed for drugs with high IOV (Karlsson and Sheiner, 1993; Wallin et al., 2010; Holford and Buclin, 2012; Liefaard and Chen, 2015). An approach describing the safe and effective variability (SEV) has been proposed to evaluate when TDM is not feasible (Holford and Buclin, 2012). However, MIPD can be used to overcome the challenge of high IOV by optimizing a dose based on individually observed plasma concentrations, together with information from a POPPK, PBPK, or PKPD model to ensure efficacious individual dosages.

Rifampicin is one of the core-drugs in the first-line treatment of drug-susceptible tuberculosis (TB) (WHO, 2017). With the currently recommended dosage of 10 mg/kg once daily (WHO, 2017), a substantial amount of patients do not reach sufficient drug exposure, which has been shown to result in treatment failure, development of drug resistance, and disease relapse (Pasipanodya et al., 2013; Alsultan and Peloquin, 2014; Ramachandran et al., 2019). Therefore, higher doses of rifampicin have been studied and were shown to lead to higher efficacy than the current recommended dosage (Svensson et al., 2018b), while still being safe (Boeree et al., 2015). It is crucial to ensure adequate plasma drug concentrations in every patient and reduce the variability between patients, in order to maximize the clinical efficacy and decrease the probability of safety related issues. Furthermore, rifampicin is a drug known to exhibit a variety of PK complexities, including induction of its own metabolism (auto-induction), dose-dependent bioavailability, and concentration-dependent clearance (Smythe et al., 2012; Chirehwa et al., 2016; Svensson et al., 2018a). In addition, there is a moderate IIV (IIV in AUC_0–24h_ of 25.4%) (Al-Sallami et al., 2014), and high IOV (IOV in AUC_0–24h_ of 25.8%) in the PK, which creates difficulties in performing individual dosing. Rifampicin IIV leads to variation in exposure between patients given the same dose, even if weight-based dosing is used (Susanto et al., 2019). While some of the variability between patients can be explained with patient characteristics such as HIV co-infection or sex (McIlleron et al., 2006), differences in drug formulations (McIlleron et al., 2006), pharmacogenetic factors (Weiner et al., 2010; Chigutsa et al., 2011), and variability in drug absorption, depending on concomitant food intake (Polasa and Krishnaswamy, 1983; Zent and Smith, 1995; Peloquin et al., 1999), some remain unexplained and are expressed as IIV.

Svensson et al. developed a MIPD approach for dose individualization of high-dose rifampicin, able to handle all the above mentioned complexities in PK (Svensson et al., 2019), based on a POPPK model (Svensson et al., 2018a) that has been shown to be best suitable for MIPD of rifampicin (van Beek et al., 2019). In this approach, an average exposure (AUC_0–24h_ at steady state: 235 h mg/L) corresponding to a high dose of 35 mg/kg in the PanACEA HIGHRIF1 trial, an open-label phase II multiple dose-rising trial registered at www.clinicaltrials.gov (NCT01392911) (Boeree et al., 2015; Svensson et al., 2018a; Svensson et al., 2019) is targeted, a dose that was found to be safe, while still resulting in high efficacy.

In this simulation study we investigated the magnitude of IOV compared to IIV in rifampicin PK on an exposure level, the impact of not acknowledging IOV when performing MIPD, the performance of the proposed MIPD approach, and the number of sampling occasions needed to predict individual doses accurately and precisely.

## Material and Methods

### Simulations

The simulations were performed based on the covariate distribution of the PanACEA HIGHRIF1 Phase II study population (Boeree et al., 2015; Svensson et al., 2018a), which consisted of 83 adult patients from Cape Town, South Africa, with drug-susceptible pulmonary TB. No ethics approval from an ethics committee and written informed consent from participants had to be obtained, since all data was simulated, and therefore no personal data was handled. The distribution of the demographics; sex, bodyweight (WT), and fat-free mass (FFM) in the simulated population was obtained by sampling from the empirical covariate distribution of the HIGHRIF1 study population (**Table 1**), taking into account the correlation between sex, WT, and FFM. First, sex was assigned to the simulated patients, according to the original distribution (71.1% male patients and 28.9% female patients). WT was then assigned for each virtual patient based on the WT distribution in either male or female patients in the original study population. Finally, FFM values were obtained separately for male and female simulated patients from two correlation functions empirically derived from the original data, describing the relationship between WT and FFM for male patients (Eq. 1) and female patients (Eq. 2).

(Eq. 1) $$FFM_{male_{i}}=2.541\times WT_{i}^{0.728}$$

(Eq. 2) $$FFM_{female_{i}}=2.496\times WT_{i}^{0.669}$$

**Table 1:** Demographics and covariates of the PanACEA HIGHRIF1 study population (Boeree et al., 2015; Svensson et al., 2018a) used for the simulations.

	All subjects	Male patients	Female patients
**N**	83	59	24
**WT (kg)** **Mean (range)**	55.1 (40.2–84.2)	55.8 (40.7–74.0)	53.8 (40.2–84.2)
**FFM (kg)** **Mean (range)**	40.0 (28.5–57.9)	47.4 (37.6–57.9)	35.7 (28.5–47.8)

WT, bodyweight, FFM, fat-free mass.

Rifampicin plasma concentrations were simulated (given covariates and doses) for 1,000 virtual patients per dose level (10, 20, 25, 30, and 35 mg/kg), following a sparse sampling schedule including samples pre-dose (5 min before dose) and at 2 and 4 h post-dose relating to the suggested sampling scheme by van Beek et al. (2019). Samples were taken at three sampling occasions (days 1, 7, and 14). A sampling occasion has been defined as a visit during which plasma drug concentrations were collected.

### Population Pharmacokinetic Model

All simulations of rifampicin plasma concentrations were performed from a previously published POPPK model developed by Svensson et al. (2018a). The model consists of a one-compartment disposition model and elements accounting for rifampicin dose-dependent bioavailability, auto-induction, and concentration-dependent clearance. Rifampicin is known to induce its own metabolism through the activation of nuclear pregnane X receptors (PXRs), which leads to a reduction of rifampicin plasma concentration over time (auto-induction) (Chen and Raymond, 2006). Drug absorption was described by a transit compartment model with the parameters mean transit time (MTT), number of transit compartments (NN), and a transfer rate between transit compartments (k_tr_), describing the delay in absorption. To account for the dose-dependent bioavailability, an E_max_ function was implemented (Eq. 3) describing a nonlinear increase in bioavailability with increasing doses above 450 mg such as:

(Eq. 3) $$F=F_{450}\times \left(1+\frac{F_{max}\times \left(Dose-450\right)}{ED_{50}+\left(Dose-450\right)}\right)$$

where F_450_ is the bioavailability (F) at a dose of 450 mg, which was assumed to be 1 due to the lack of data for doses below 450 mg, F_max_ is the maximal increase in F with increasing doses, and ED_50_ is the dose at which the increase in F is half-maximal. The auto-induction was characterized by an enzyme turnover model developed by Smythe et al. (2012). In order to be able to distinguish between auto-induction and capacity-limited elimination, the enzyme turnover model by Smythe et al. was implemented without structural modifications. The concentration-dependent apparent clearance (CL/F) was described by a Michaelis-Menten relationship (Eq. 4),

(Eq. 4) $$CL/F=\;\frac{V_{max}}{k_{m+C_{p}}}$$

where V_max_ is the maximal elimination rate, and k_m_ is the plasma concentration (C_p_) at which the elimination rate is half of V_max_. This capacity-limited elimination is assumed to occur due to saturable efflux transporters in the bile (Acocella, 1978). For description of variability in PK, IIV on the parameters V_max_, k_m_, V, k_a_, MTT and NN, and IOV on the parameters k_m_, k_a_, MTT, and F were incorporated in the model. The residual variability was described using an additive error model on log-scale (Svensson et al., 2018a). Data below lower limit of quantification (LLOQ), both during model building and generated from simulation, were handled using the M3 method (Beal, 2001).

### Model-Informed Precision Dosing Algorithm

**Figure 1:**
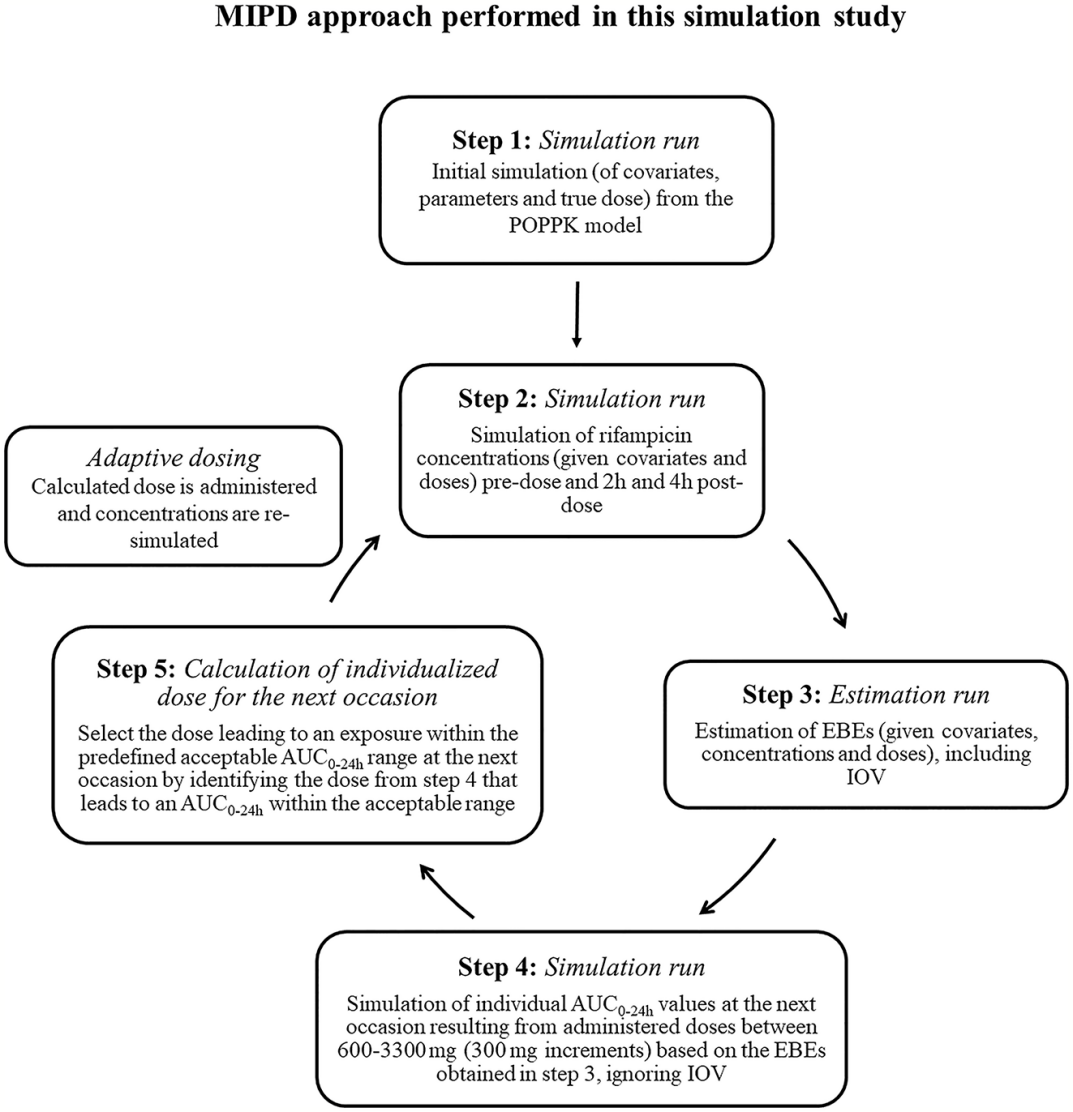
Workflow of the model-informed precision dosing (MIPD) approach performed in this simulation based study. AUC_0–24h_, area under the plasma concentration-time curve from time zero up to 24 h; PK: pharmacokinetic; IOV: inter-occasion variability.

**Figure 2:**
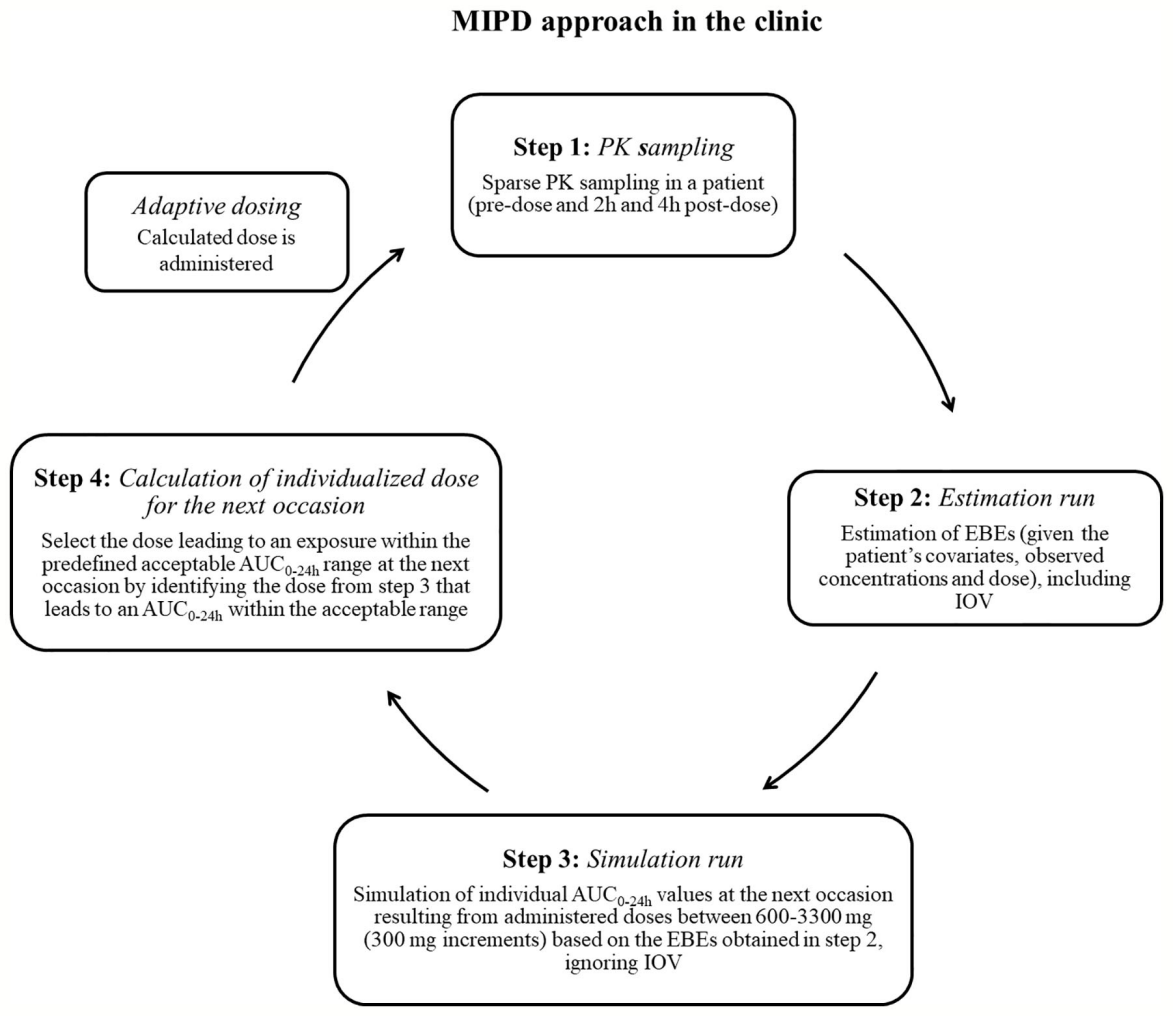
Suggested workflow for performance of the model-informed precision dosing (MIPD) approach in the clinic. AUC_0–24h_, area under the plasma concentration-time curve from time zero up to 24 h; PK: pharmacokinetic; IOV: inter-occasion variability.

To mimic a MIPD scenario in the clinic, a rifampicin MIPD algorithm (**Figures 1** and **2**) based on a method developed by Svensson et al. (2019) was utilized with two modifications. The modifications made included; the addition of the NOABORT option during the estimation step and the inclusion of additional ETA values to describe additional sampling occasions. In order to evaluate the performance of different MIPD scenarios, the true dose was obtained for each individual patient for comparison. The true dose was calculated based on the true parameter values which were derived in an initial simulation from the POPPK model (see **Figure 1**). The MIPD predicted dose for each individual was determined using the Empirical Bayes estimates (EBEs) of PK information given covariates and concentrations derived from the sparse sampling (see *Simulations* and **Figure 1**). For the first sampling occasion (day 1), only plasma drug concentrations from this occasion were included whereas for the following occasions (days 7 and 14), the accumulated plasma drug concentrations for each individual were used to derive the EBEs, mimicking a real clinical setting. The dose on day 1 was determined with the covariate WT, since in the clinic rifampicin is often dosed per kilogram WT. Depending on the simulated subject’s WT, a dose was assigned according to the weight-bands used in the HIGHRIF1 study (Boeree et al., 2015). The dose at day 7 was determined based on the EBEs derived from covariate information and concentrations from sampling at day 1 and the dose at day 14 was determined based on the EBEs derived from covariate information and concentrations from sampling at day 1 and 7. A simulation step occurred in between each estimation step given the new dose. This means that plasma concentrations were simulated given the dose determined after the first sampling occasion (day 1) in order to estimate the EBEs at day 7. For estimation of EBEs at day 14, plasma concentrations were simulated given the dose determined on day 7. In order to predict individual doses using the MIPD approach, first the EBEs for each individual were derived based on covariate information and observed plasma concentrations from the sparse sampling, either including information from one, two, or three sampling occasions. IOV was included when estimating EBEs (Abrantes et al., 2019). In order to improve the accuracy in EBE estimation, the MCETA option in NONMEM was applied (MCETA=100). MCETA is a setting for maximum a posteriori estimation, i.e. η-optimization. By default, the initial value for all η-values is zero. Setting MCETA to a larger number than 1 allows additional η-values to be tested. As additional initial η-values the η-values from the previous iteration, as well as random samples taken from a normal distribution with the variance Ω, will be evaluated. The initial η-values resulting in the lowest objective function value (OFV) will be chosen as initial values (Beal et al., 1989). Based on the so obtained EBEs, area under the plasma concentration-time curve from time zero up to 24 h (AUC_0–24h_) values following administered doses of 600–3,300 mg (300 mg increments) were predicted for the next occasion for each individual. As suggested by Abrantes et al. (2019), IOV was not included in these predictions. Thereafter, a R code (**Supplementary appendix S2 R code**) was used to select the dose corresponding to the simulated AUC_0–24h_ value closest to the target AUC_0–24h_. The target AUC_0–24h_ and its acceptable range have previously been defined by Svensson et al. (2019) (see **Table 2**). The target AUC_0–24h_ (with its accompanying acceptable range) varies with time due to the auto-induction of rifampicin elimination. In order to empirically mimic the time variation in exposure, a time-varying target has been determined according to the observed typical AUC_0–24h_ seen in patients depending on the day after first dose, up to day 56 where a fully induced state has been reached (Svensson et al., 2019). The acceptable range around the target reflects the observed variability in exposure, including that the drug is only available in tablet strengths of 300 mg increments. As suggested by Svensson et al. (2019), the acceptable range was centered around 35 mg/kg rifampicin. The mid‐point between the AUC_0–24h_ predicted for the typical individual following a dose of 30 and 35 mg/kg represented the lower limit of the interval and the mid‐point between the AUC_0–24h_ for 35 and 40 mg/kg the upper limit. In cases where more than one of the predicted doses would lead to an exposure within the acceptable range around the target AUC_0–24h_, the lowest dose that led to an exposure within the acceptable range was chosen. Due to the nature of the dosing algorithm, it is possible that more than one simulated AUC_0–24h_ value following administered doses of 600–3,300 mg would fall within the acceptable range of AUC_0–24h_. For example, in one individual the AUC_0–24h_ following a dose of 1,500 mg is 190 h mg/L and the AUC_0–24h_ following a dose of 1,800 mg is predicted to be 222 h mg/L. Both AUC_0–24h_ values would fall within the acceptable range of 189–224 h mg/L at day 14 after first dose. Due to safety reasons, the lower dose that led to an exposure within the acceptable range of AUC_0–24h_ (in this example 1,500 mg) was selected. In cases where none of the predicted doses led to an exposure within the acceptable range of AUC_0–24h_, the dose closest to the acceptable range of AUC_0–24h_ was selected. To account for the IOV in PK, a previously suggested approach (Wicha and Hennig, 2018; Abrantes et al., 2019; Svensson et al., 2019) was applied, where IOV is included in the EBE estimation, but ignored in the individual parameter, here AUC_0–24h_, used to calculate the forecasted dose.

**Table 2:** Bayesian acceptable ranges of AUC_0–24h_ used for dose individualization (Svensson et al., 2019).

Time after first dose (days)	AUC_0–24h_ (h. mg/L)
1	342–408
7	217–259
14	189–224
28	182–215
56	181–214

AUC_0–24h_, area under the plasma concentration-time curve from time zero up to 24 h.

### Illustration and Magnitude of IOV in the PK of Rifampicin on an Individual Level

In order to illustrate the phenomenon IOV and to investigate the magnitude of IOV in rifampicin PK on an exposure level, the steady-state AUC_0–24h_ for the typical patient were simulated at 1,000 repeated sampling occasions and the 95% PI was computed. Each sampling occasion here represented a visit where PK samples were taken in steady state. The number of simulations was set to 1,000 in order to achieve an accurate value for the 95% PI. The 95% PI was calculated by computing the 2.5^th^ and the 97.5^th^ percentiles of the distribution of the simulated AUC_0–24h_ values. These percentiles are given by the (N + 1)α^th^ and (N + 1)(1 − α)^th^ elements of the ordered AUC_0–24h_ values, where α is 0.025 and N the number of simulated AUC_0–24h_ values. The typical patient was a male patient with a WT of 53.9 kg and a FFM of 44.6 kg, receiving a dose of 35 mg/kg (1,800 mg), and steady-state was beyond day 24 after the first dose (Svensson et al., 2018a). The simulations were performed with only including IOV on the PK parameters, hence not including residual error or IIV in the simulations. The 95% PI of these simulated AUC_0–24h_ values visualizes the magnitude of IOV in the typical individual of the HIGHRIF1 study population. The magnitude of IOV has been expressed as described in equation 5.

(Eq. 5) $$IOV\;(\%)=\;\sqrt[{2}]{\;\frac{1}{N}\times \Sigma_{i=1}^{N}{\left(\text{ln}{\left(AUC\right)}_{i}-\mu_{\ln(AUC)}\right)}^{2}}\times 100\%$$

To illustrate IOV in a figure, 20 randomly chosen simulated sampling occasions out of the 1,000 occasions were selected and the AUC_0–24h_ versus occasion was plotted.

### Magnitude of IIV in Rifampicin PK

The magnitude of IIV in rifampicin PK was investigated by simulating plasma concentrations for 1,000 patients receiving a dose of 35 mg/kg rifampicin at steady state and thereafter deriving the AUC_0–24h_. The simulations were performed with only including IIV on the PK parameters, hence not including residual error or IOV in the simulations. The 95% PI of these simulated AUC_0–24h_ values illustrates the magnitude of IIV in the HIGHRIF1 study population. The magnitude of IIV has been expressed as described in equation 6.

(Eq. 6) $$IIV\;(\%)=\;\sqrt[{2}]{\;\frac{1}{N}\times \Sigma_{i=1}^{N}{\left(\text{ln}{\left(AUC\right)}_{i}-\mu_{\ln(AUC)}\right)}^{2}}\times 100\%$$

### Predicted Exposure in a Population Accounting or Not Accounting for IOV

In order to explore the drug exposure on a population level accounting or not accounting for IOV, the AUC_0–24h_ distribution within the population was derived by simulating rifampicin plasma concentrations for 1,000 patients per dose level (10, 20, 25, 30, and 35 mg/kg) at days 1, 7, and 14, either including or neglecting IOV in the parameters k_m_, k_a_, MTT, and F. When IOV was neglected in the simulations, it was fixed to zero in the parameters. Including IOV in the simulations, represents the real observed exposure in patients.

### Evaluating the Predictive Performance of MIPD Incorporating or Ignoring IOV

In order to explore the impact of not accounting for IOV during model building on the performance of MIPD, a separate miss-specified POPPK model without IOV was developed. The POPPK model ignoring IOV was developed by fixing all IOV parameters to zero in the original model, whereas all remaining population typical PK parameters, IIV, and residual variability parameters were re-estimated using the original dataset used to develop the POPPK model. This led to technical issues, and thus the PsN function –parallel_retries to alter the set of initial estimates by 10%, and the NONMEM option NOABORT were utilized, which resulted in better numerical stability and a final model which described the data well. The OFV for this model was 1,727.4 points higher than the OFV of the original final POPPK model. The code for the POPPK model ignoring IOV is provided in the supplementary material (**Supplementary Appendix S1 Model code**). The POPPK model ignoring IOV was then used to perform MIPD, and the prediction error was compared to the prediction error when performing MIPD based on the original POPPK model, which included IOV. The individual prediction error was computed for the MIPD approach accounting for and the approach ignoring IOV in the underlying model as:

(Eq. 7) $$\mathrm{Individual}\;\mathrm{Prediction}\;\mathrm{Error}\;\left(\%\right)=\;\frac{\mathrm{Dos}e_{\mathrm{MIP}D_{i}}-{\mathrm{Dose}}_{{\mathrm{True}}_{i}}}{{\mathrm{Dose}}_{\mathrm{Tru}e_{i}}}\times 100\%$$

where Dose_MIPDi_ is the individually forecasted dose at the next occasion, calculated based on EBEs derived from a sparse sampling (pre-dose, 2 and 4 h post-dose) using information from two sampling occasions (days 1 and 7) either estimated from the original or the re-estimated POPPK model without IOV, and Dose_Truei_ was the dose calculated based on the true individual PK parameters. The 12.5^th^ and 87.5^th^ percentiles (75% PI) of the distribution of individual prediction errors were then presented in a boxplot. To evaluate the performance of the MIPD scenario including IOV in the underlying model, the distribution of predicted doses was compared to the distribution of true doses.

### Number of Sampling Occasions Needed to Predict the Dose Using a MIPD Approach

In order to assess the number of sampling occasions needed to predict the dose using MIPD, the bias [mean absolute percentage error (MAPE)] (Eq. 8) and imprecision [relative root mean square error (rRMSE)] (Eq. 9) in individual dose predictions using one, two, or three sampling occasions for the EBE estimations was evaluated. In equations 8 and 9, DOSE_MIPDi_ is the dose calculated based on the MIPD approach with EBEs derived from a sparse sampling (pre-dose, 2 and 4 h post-dose samples) either including information from one (day 1), two (day 1, 7), or three (day 1, 7, 14) occasions. When more than one sampling occasion was included in the estimation of EBEs, the dose was updated after each sample. DOSE_Truei_ is the dose calculated based on the true individual PK parameters. The difference in bias and imprecision between the different numbers of occasions was assessed. Statistical significance was evaluated by computing the 95% confidence interval for the difference between two occasions and judged to be statistically significant if it did not include zero (associated p-value = 0.05) (Sheiner and Beal, 1981).

(Eq. 8) $$\mathrm{MAPE}=100\%\times \frac{1}{N}\sum_{i}\frac{\left|DOSE_{MIPD_{i}}-DOSE_{True_{i}}\right|}{DOSE_{True_{i}}}$$

(Eq. 9) $$rRMSE=100\%\times \sqrt{\frac{1}{N}\sum_{i}\frac{{\left(DOSE_{MIPD_{i}}-DOSE_{True_{i}}\right)}^{2}}{{\left(DOSE_{True_{i}}\right)}^{2}}}$$

### Softwares

The re-estimation and all simulations were carried out in NONMEM 7.30 (Icon Development Solutions, Hanover, MD, USA) (Beal et al., 1989), assisted by PsN 4.9.1 (Department of Pharmaceutical Biosciences, Uppsala University, Uppsala, Sweden) (Keizer et al., 2013). Estimation of the parameters was performed using a Laplacian first-order conditional estimation method with interaction. Data management and visualization were performed in R statistical software version 3.6.1 (R Foundation for Statistical Computing, Vienna, Austria) (R Core Team, 2015). The “ggplot2” package was used for graphical evaluation (Wickham, 2016).

## Results

### Simulations

The mean WT ± standard deviation in the original and subsequently simulated population was 53.8 ± 10.7 kg (range 40.2–84.2 kg) for female patients and 55.8 ± 7.4 kg (range 40.7–74.0 kg) for male patients.

### Illustration and Magnitude of IOV in the PK of Rifampicin on an Individual Level

**Figure 3:**
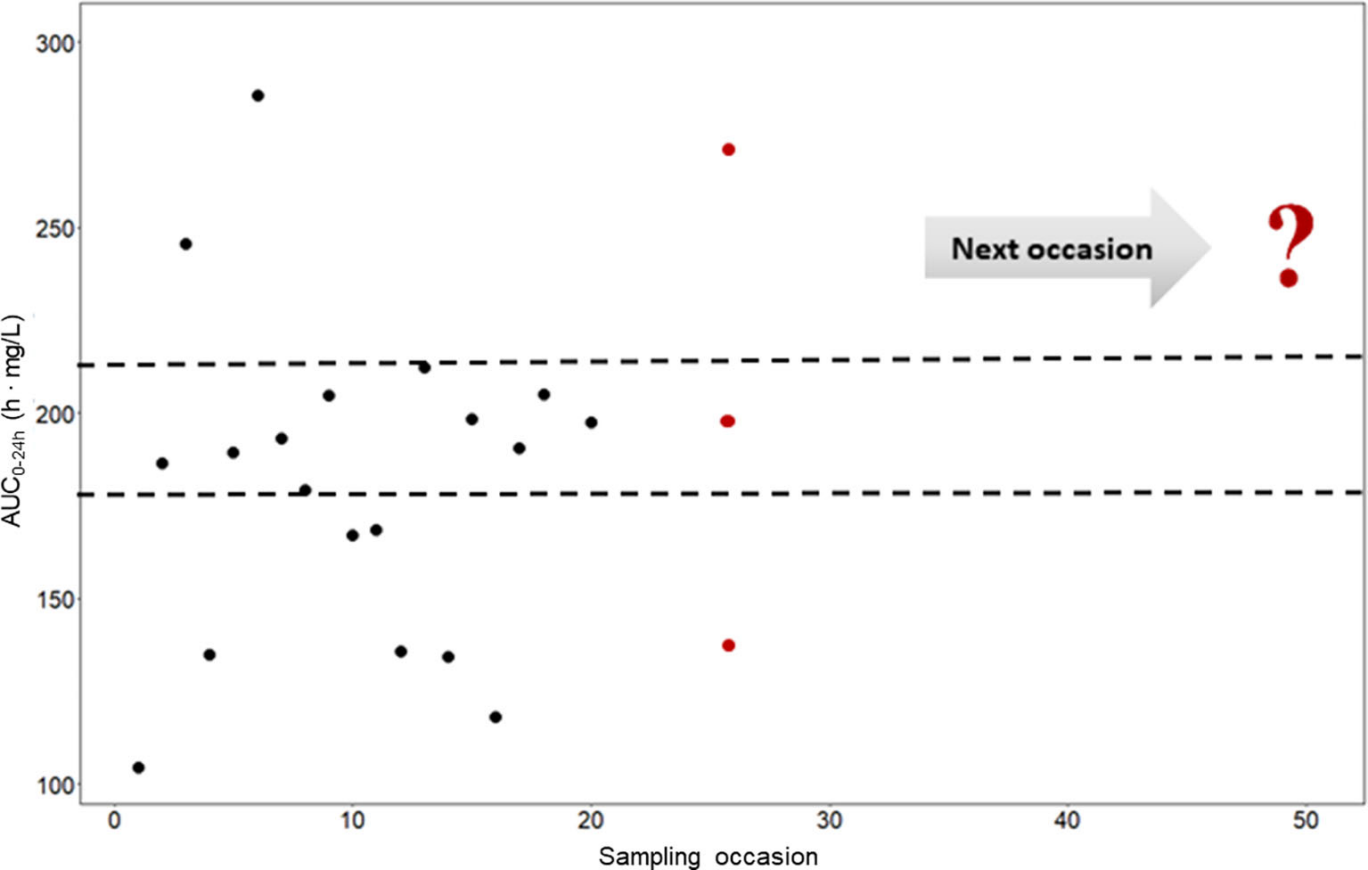
Predicted AUC_0–24h_ (h • mg/L) in a typical patient at different sampling occasions at steady state (beyond day 24) after administration of 35 mg/kg (1,800 mg) rifampicin daily (black filled circles). The different predicted exposures are due to inter-occasion variability (IOV). The dashed line represents the Bayesian acceptable range of AUC_0–24h_ at steady-state as suggested by Svensson et al. (2019). The difficulties in decision making using only observed exposure is illustrated at the 25^th^ sampling occasion (red filled circles). There are three possible decisions; no dose change (within acceptable range), decrease the dose (exposure above acceptable range), or increase the dose (exposure below acceptable range). The question mark illustrates the difficulties in decision making using a non-model based approach which ignores IOV. Using a MIPD approach, which handles the IOV and the wide range in individual exposures, a correct dose can be predicted. AUC_0–24h_, area under the plasma concentration-time curve from time zero up to 24 h.

**Figure 4:**
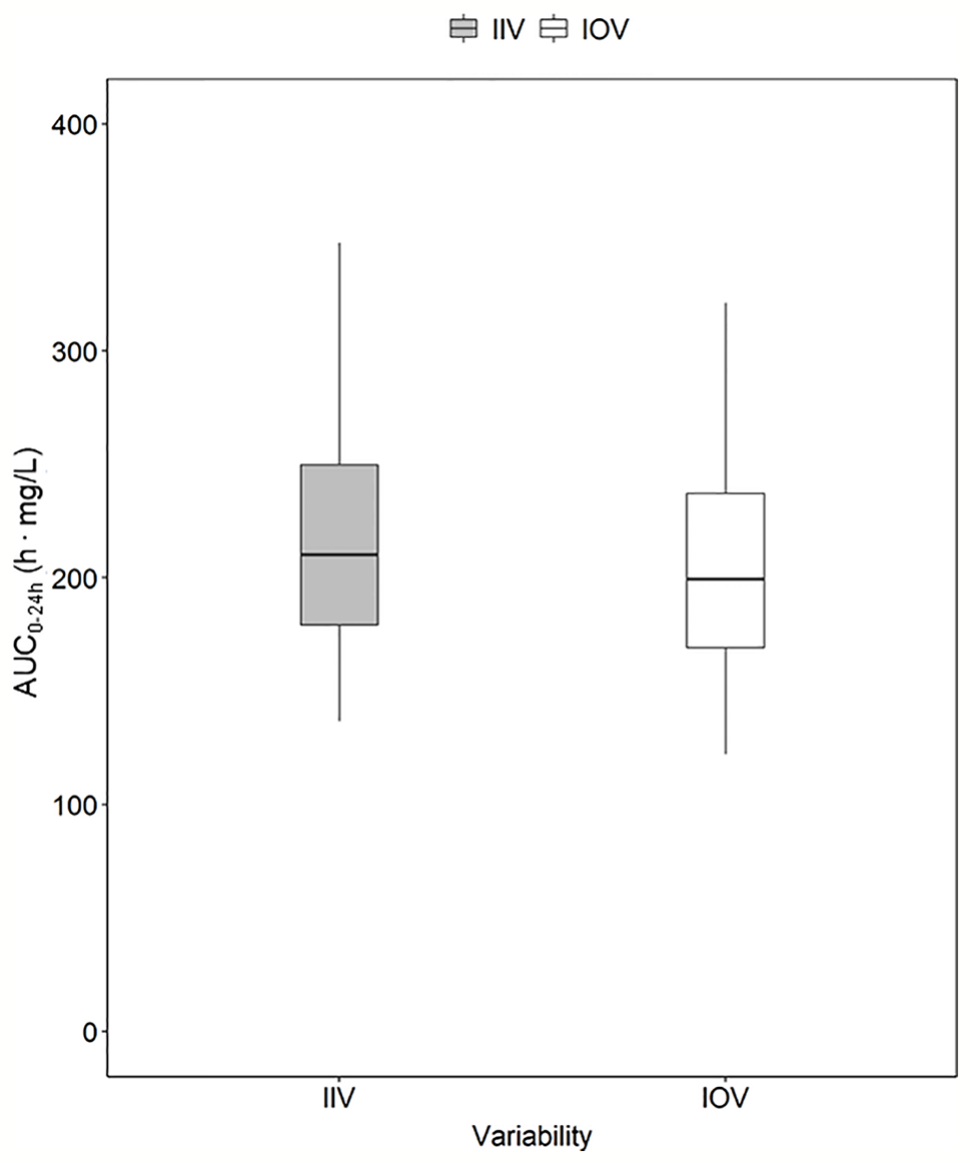
Magnitude of inter-individual variability (IIV, grey) and magnitude of inter-occasion variability (IOV, white) in AUC_0–24h_ (h. mg/L). The AUC_0–24h_ distribution between subjects due to IIV shown in this plot was derived by simulating the AUC_0–24h_ for 1,000 individuals receiving a dose of 1,800 mg with only IIV at steady state (day 24). The AUC_0–24h_ distribution within one individual between different sampling occasions due to IOV was derived by simulating the AUC_0–24h_ for the typical patient receiving a dose of 1,800 mg at 1,000 sampling occasions with only IOV at steady state (beyond day 24). The box range represents the 50% prediction interval (PI) and the whiskers the 2.5^th^ and 97.5^th^ percentile. AUC_0–24h_, area under the plasma concentration-time curve from time zero up to 24 h.

To investigate and demonstrate the magnitude of IOV in rifampicin PK, and to illustrate the difficulties in predicting the next dose for a drug with high IOV, the AUC_0–24h_ at 1,000 different sampling occasions at steady state following a dose of 1,800 mg (35 mg/kg) was simulated for the typical individual and is illustrated in **Figure 3**, showing 20 randomly selected sampling occasions. Since IOV occurs at random, it is not possible to foresee what the exposure at the next occasion will be. As shown with the red filled dots in **Figure 3**, the forecasted AUC_0–24h_ at the next occasion for the typical patient at 1,800 mg (35 mg/kg) could be anywhere within the simulated range, which ranged from 77.4 h•mg/L to 537.0 h•mg/L (95% PI: 122.2 h mg/L–331.2 h mg/L, n = 1,000), i.e. a 270% range in exposure within one individual depending on the dosing occasion (**Figure 4**). The magnitude of IOV in exposure (AUC_0–24h_) was computed to be 25.8%.

### Magnitude of IIV in Rifampicin PK

In order to explore the magnitude of IIV in rifampicin PK, i.e. the variability between patients in exposure, AUC_0–24h_ was obtained. The 95% PI of the 1,000 simulated AUC_0–24h_ values ranged from 136.9 h. mg/L–369.7 h. mg/L, meaning that the exposure of patients receiving the same dose, even if a weight-based dosing is applied, can be up to 270% different (**Figure 4**). The magnitude of IIV in exposure (AUC_0–24h_) was computed to be 25.4%. **Figure 4** shows the comparison of IIV and IOV in AUC_0–24h_ at 35 mg/kg.

### Predicted Exposure in a Population Accounting or Not Accounting for IOV

**Figure 5:**
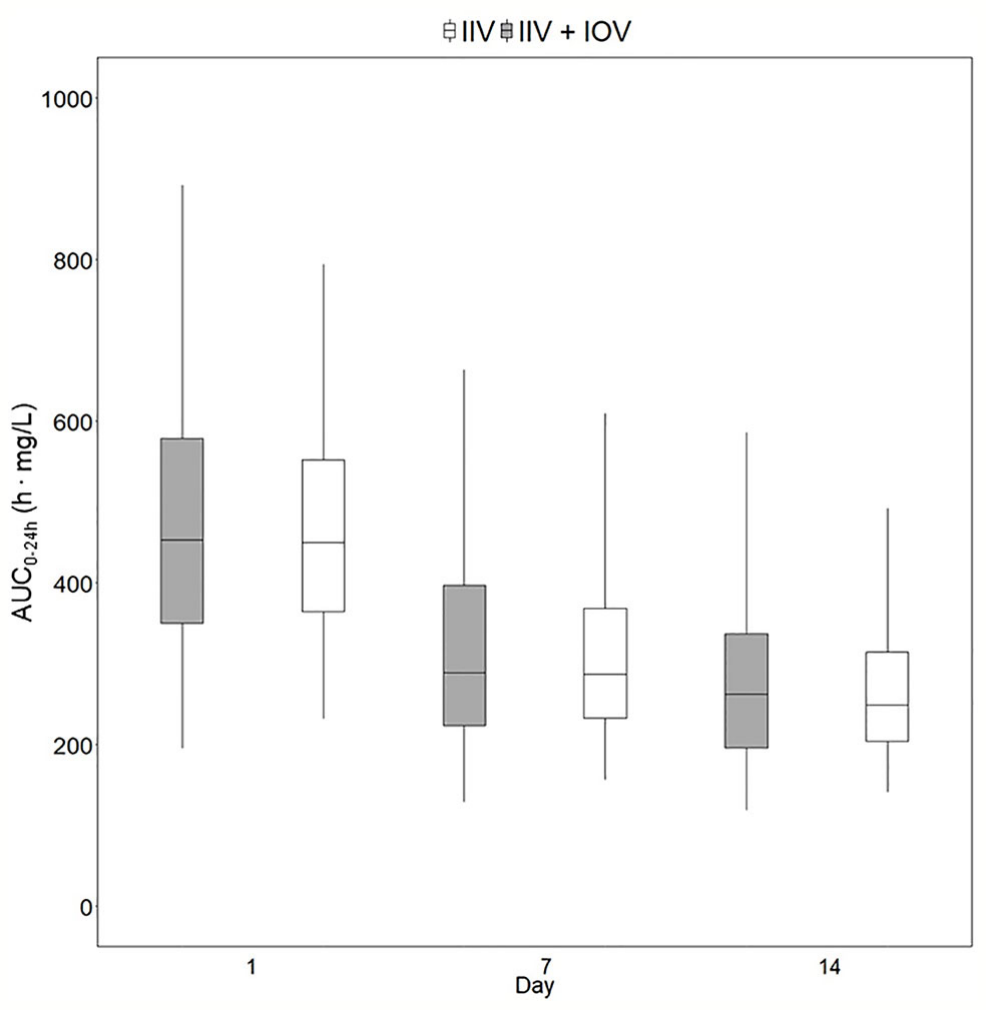
Predicted exposure in a population including or not including inter-occasion variability (IOV). Distribution of AUC_0–24h_ (h. mg/L) in 1,000 virtual patients receiving a dose of 35 mg/kg once daily, simulated including only inter-individual variability (IIV, white) or including IIV and IOV (grey). Including IOV in the simulations represents the real observed range in exposure in TB patients. The box range represents the 50% prediction interval (PI) and the whiskers the 2.5^th^ and 97.5^th^ percentile. AUC_0–24h_, area under the plasma concentration-time curve from time zero up to 24 h.

The AUC_0–24h_ distribution in the different dose groups was simulated with or without IOV. When IOV was incorporated into the simulations, the AUC_0–24h_ distribution was larger for all dose groups (95% PI for a dose of 35 mg/kg: 118.9–586.7 h_._ mg/L at day 14) compared to when IOV was disregarded (95% PI for a dose of 35 mg/kg: 141.3–491.6 h_._ mg/L), as shown in **Table 3** and **Figure 5**. The median of the simulated AUC_0–24h_ values was fairly similar between simulations including both IIV and IOV (261.9 h_._ mg/L) and simulations with only IIV (248.7 h_._ mg/L). The median of the deviation in AUC_0–24h_ between simulating with and without IOV for each individual at day 14 (dose: 35 mg/kg) was 7.7 h_._ mg/L (95% PI: −260 to 305 h_._ mg/L) (**Table 3**).

**Table 3:** Simulated AUC_0–24h_ (h • mg/L) at day 14 after daily dosing, with only inter-individual variability (IIV) or with IIV + inter-occasion variability (IOV), as well as median individual deviation in simulated AUC_0–24h_ (h • mg/L) between simulating with only IIV or with IIV + IOV.

Dose group	Median AUC_0–24h_ (h • mg/L) (95% PI)		Median individual deviation (h • mg/L) (95% PI)
	IIV	IOV + IIV	
10 mg/kg	39.9 (18.8 to 81.6)	39.3 (16.4 to 93.6)	0.6 (−40 to 48)
20 mg/kg	120.3 (68.0 to 230.5)	123.5 (59.4 to 266.7)	1.9 (−12 to 36)
25 mg/kg	151.4 (82.8 to 297.9)	155.3 (72.5 to 348.4)	2.7 (−152 to 181)
30 mg/kg	194.3 (107.2 to 382.3)	202.6 (91.0 to 452.5)	5.2 (−197 to 231)
35 mg/kg	248.7 (141.3 to 491.6)	261.9 (118.9 to 586.7)	7.7 (−260 to 305)

In **Figure 3**, the simulated AUC_0–24h_ in 1,000 patients for a dose of 35 mg/kg at days 1, 7, and 14 is illustrated.

PI, prediction interval; IIV, inter-individual variability; IOV, inter-occasion variability.

### Evaluating the Predictive Performance of MIPD Incorporating or Ignoring IOV

**Figure 6:**
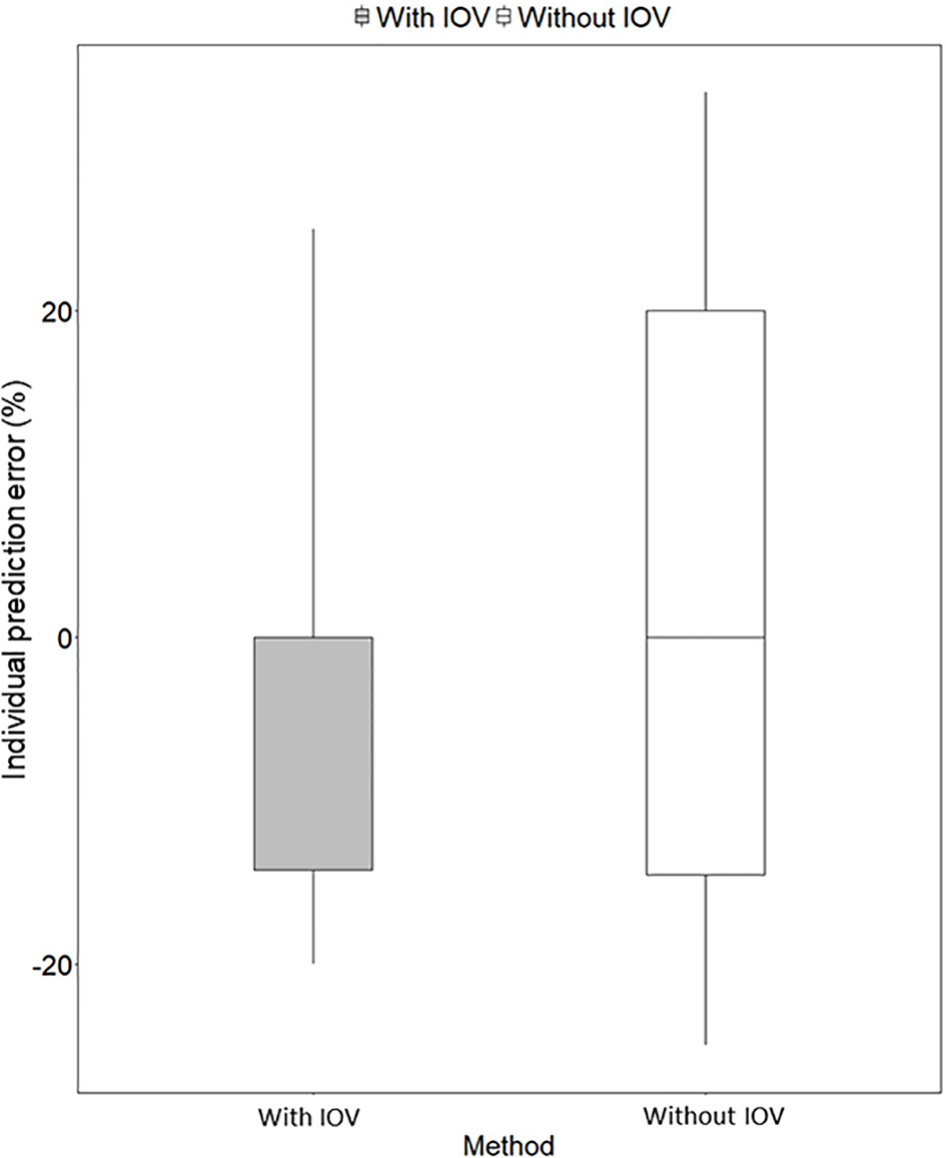
Individual prediction error (%) for predicting the next dose with a MIPD approach based on a population pharmacokinetic (POPPK) model without inter-occasion variability (IOV, white box) compared to a POPPK model with IOV (grey box). The boxplot body represents the 75% prediction interval (PI) and the whiskers the 2.5^th^ and 97.5^th^ percentile. The median of the individual prediction error is shown by the black line and is 0% for both approaches. In the approach incorporating IOV, a skewed distribution, skewed to lower doses is predicted, which is favorable from a safety point of view.

**Figure 7:**
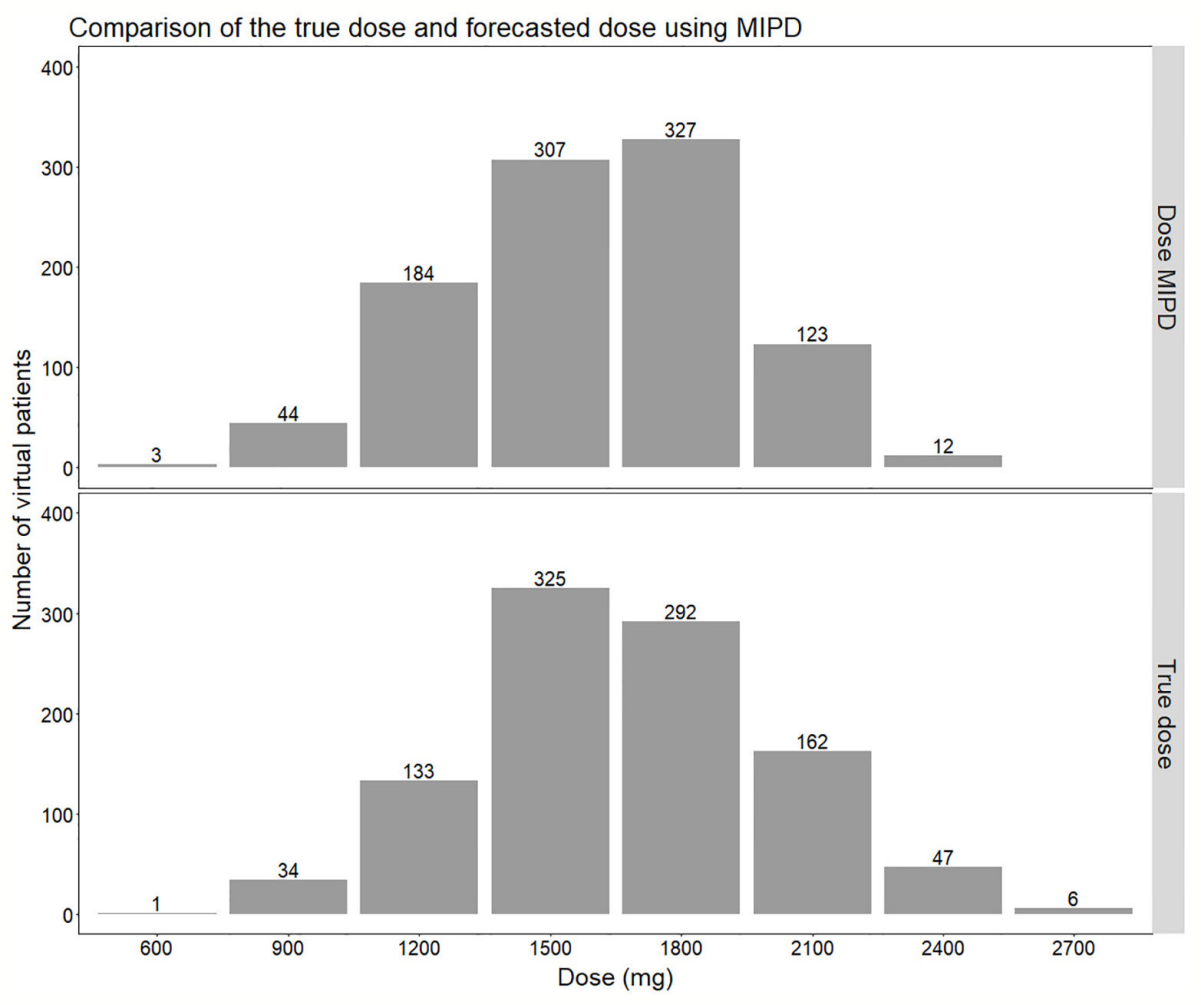
Comparison of the distribution of doses forecasted using the true individual PK parameters (lower panel) compared to using Empirical Bayes Estimates (EBEs) from a model-informed precision dosing (MIPD) scenario including IOV (Inter-occasion variability) (upper panel). In the MIPD approach, doses were predicted including information from two sampling occasions 7 d apart.

Re-estimation of the POPPK model without IOV, i.e. IOV fixed to zero, resulted in an increase of the variability components; residual error and IIV, apart from IIV in the parameter k_a_. When IOV was ignored, the residual error increased from 23.6% to 56.9%, IIV in k_m_ increased from 35.8% to 80.6% and IIV in MTT increased from 38.2% to 58.8%. However, the IIV in k_a_ decreased from 33.8% to 28.7% when IOV was omitted. The final parameter estimates for the rifampicin POPPK model for high dose rifampicin when ignoring IOV are provided in **Table 4**. The 75^th^ percentile of the individual prediction errors was greater using a MIPD approach which ignored IOV (median: 0%, 75% PI: −14.6% to 20.0%) compared to a MIPD approach acknowledging IOV in the underlying model (median: 0%, 75% PI: −14.6% to 0.0%) (**Figure 6**). The difference in individual prediction error between both approaches was larger for a 75% PI compared to a 95% PI. Therefore, the results are presented using the 75% PI. A MIPD approach incorporating IOV and taking into account information from two sampling occasions (days 1 and 7) performed well with respect to forecasting the dose at the next dosing occasion. In **Figure 7**, the distribution of predicted doses derived from the MIPD approach compared to the true doses is illustrated.

**Table 4:** Final model parameter estimates of the original population pharmacokinetic (POPPK) model developed by Svensson et al. (2018a) compared to final parameter estimates of the re-estimated POPPK model without inter-occasion variability (IOV).

Parameter	Description	Estimates original model	Estimates model without IOV (RSE [%])
V_max_ (mg/h/70 kg)	Maximal elimination rate	525	309.8 (19.9%)
k_m_ (mg/L)	Concentration at which half of the elimination is reached	35.3	15.8 (19.5%)
V (L/70 kg)	Volume of distribution	87.2	93.6 (8.0%)
k_a_ (h^−1^)	Absorption rate constant	1.77	2.4 (43.2%)
MTT (h)	Mean transit time	0.51	0.81 (16.0%)
NN	Number of transit compartments	23.8	7.6 (25.7%)
E_max_	Maximal increase in enzyme production rate	1.16	1.2 (11.6%)
EC_50_ (mg/L)	Concentration at which half E_max_ is reached	0.0699	0.053 (125.6%)
k_ENZ_ (h^−1^)	First-order rate constant for enzyme degradation and formation	0.00603	0.0053 (25.0%)
F_max_	Maximal increase in bioavailability with doses above 450 mg	0.504	0.40 (33.9%)
ED_50_ (mg)	The dose at which half F_max_ is reached	67.0	17.4 (374.4%)
IIV V_max_ (%)	Inter-individual variability in V_max_	30.0	55.2 (17.7%)
IIV k_m_ (%)	Inter-individual variability in k_m_	35.8	80.6 (13.8%)
IIV V (%)	Inter-individual variability in V	7.86	8.6 (64.4%)
IIV k_a_ (%)	Inter-individual variability in k_a_	33.8	28.7 (174.8%)
IIV MTT (%)	Inter-individual variability in MTT	38.2	58.8 (18.8%)
IIV NN (%)	Inter-individual variability in NN	77.9	90.5 (18.3%)
IOV k_m_ (%)	Inter-occasion variability in k_m_	18.9	0 FIX
IOV k_a_ (%)	Inter-occasion variability in k_a_	31.4	0 FIX
IOV MTT (%)	Inter-occasion variability in MTT	56.4	0 FIX
IOV F (%)	Inter-occasion variability in F	15.7	0 FIX
Correlation V_max_-K_m_ (%)		38.9	59.6 (15.9%)
(%) Additive error on log scale		23.6	56.9 (0.9%)

IOV, inter-occasion variability; RSE, relative standard error reported on the approximate standard deviation scale.

### Number of Sampling Occasions Needed to Predict the Dose Using a MIPD Approach

The imprecision (rRMSE) and bias (MAPE) in predicted doses using a MIPD approach where IOV was acknowledged, decreased statistically significant (p-value < 0.05), when information from a second sampling occasion was taken into account compared to only one sampling occasion (difference in imprecision: −9.1%, bias: −7.7%). The imprecision and bias decreased however only marginally, not statistically significant, when data from a third occasion was included (difference in imprecision: −0.1%, bias: −0.1%). **Table 5** provides the imprecision (rRMSE) in dose predictions and **Table 6** shows the bias (MAPE) in dose predictions for all doses.

**Table 5:** Imprecision [relative root mean square error (rRMSE)] in individually predicted doses at the next occasion, taking information from one (day 1), two (day 1, 7), or three (day 1, 7, 14) sampling occasions using three samples (pre-dose, and 2 and 4 h post-dose) per occasion into account for Empirical Bayes Estimates (EBE) estimation.

True dose	rRMSE (%)	ΔrRMSE (%)	rRMSE (%)	ΔrRMSE (%)	rRMSE (%)	N
	One occasion	(One/two occasions)	Two occasions	(Two/three occasions)	Three occasions	
		(95% CI)		(95% CI)		
**All doses**	19.3	[−0.10 to −0.08]^*^	10.2	[0.00 to 0.00]	10.1	1,000
**600**	50.0	–	0.0	–	0.0	1
**900**	53.3	[−0.57 to −0.30]^*^	9.9	[0.00 to 0.03]	11.4	34
**1,200**	27.8	[−0.20 to −0.15]^*^	10.2	[0.00 to 0.01]	10.8	133
**1,500**	15.9	[−0.06 to −0.05]^*^	10.5	[0.00 to 0.00]	10.5	325
**1,800**	10.5	[−0.03 to −0.02]^*^	8.3	[0.00 to 0.00]	8.5	292
**2,100**	14.8	[−0.05 to −0.03]^*^	10.9	[−0.01 to 0.00]	10.3	162
**2,400**	20.0	[−0.07 to −0.04]^*^	14.6	[−0.04 to −0.02]^*^	11.4	47
**2,700**	24.4	[−0.14 to −0.04]^*^	15.7	[0.00 to 0.00]	15.7	6

rRMSE, relative root mean square error; CI, confidence interval.

*Statistically significant difference (p-value < 0.05) in rRMSE between taking information from one or two occasions, or between taking two or three occasions, into account to predict individual doses at the next occasion.

**Table 6:** Bias [mean absolute percentage error (MAPE)] in individually predicted doses at the next occasion, taking information from one (day 1), two (day 1, 7) or three (day 1, 7, 14) sampling occasions using three samples (pre-dose, and 2 and 4 h post-dose) per occasion into account for Empirical Bayes Estimates (EBE) estimation.

True dose	MAPE (%)	ΔMAPE (%)	MAPE (%)	ΔMAPE (%)	MAPE (%)	N
	One occasion	(One/two occasions)	Two occasions	(Two/three occasions)	Three occasions	
		(95% CI)		(95% CI)		
**All doses**	13.3	[−0.09 to −0.07]^*^	5.6	[−0.01 to 0.00]	5.5	1,000
**600**	50.0	–	0.0	–	0.0	1
**900**	46.1	[−0.53 to −0.33]^*^	2.9	[−0.02 to 0.04]	3.9	34
**1200**	21.1	[−0.20 to −0.14]^*^	4.1	[−0.0 to 0.02]	4.7	133
**1,500**	11.9	[−0.08 to −0.05]^*^	5.5	[−0.01 to 0.01]	5.5	325
**1,800**	6.5	[−0.03 to −0.01]^*^	4.1	[0.00 to 0.01]	4.3	292
**2100**	12.6	[−0.06 to −0.04]^*^	7.8	[−0.02 to 0.00]	7.1	162
**2400**	18.6	[−0.08 to −0.04]^*^	12.8	[−0.05 to −0.02]^*^	9.3	47
**2700**	24.1	[−0.13 to −0.06]^*^	14.8	[0.00 to 0.00]	14.8	6

MAPE, mean absolute percentage error; CI, confidence interval.

*Statistically significant difference (p-value < 0.05) in MAPE between taking information from one or two occasions, or between taking two or three occasions, into account to predict individual doses at the next occasion.

## Discussion

Traditionally, individualized dosing has been difficult for drugs with high IOV, since it is a phenomenon occurring completely at random, creating challenges in dose predictions (Karlsson and Sheiner, 1993; Wallin et al., 2010; Holford and Buclin, 2012; Liefaard and Chen, 2015). However, this work shows that MIPD can overcome these difficulties, as illustrated using rifampicin as an example drug. In this simulation study, it could be demonstrated that rifampicin has a large IOV in PK, not only in PK parameters, but also on an exposure level (AUC_0–24h_). The IOV in AUC_0–24h_ was as high as the IIV in exposure, 25.8% and 25.4%, respectively (**Figure 4**). It is often of interest to study the magnitude of IOV and IIV in secondary parameters, such as AUC, in addition to IIV and IOV in primary PK parameters which is estimated within the POPPK model, as the IOV and IIV in PK parameters do not directly translate to IIV and IOV in secondary parameters (Friberg et al., 2002; Latz et al., 2006; Dai et al., 2008). To derive IOV in secondary parameters, concentration versus time profiles based on a POPPK model with only IOV (no IIV) should be simulated for the same individual and a large number of sampling occasions (1,000) at steady state as described in this work. IIV in secondary parameters can be quantified by simulating concentration versus time profiles based on a POPPK model including only IIV for a large number of patients (1,000) at steady-state. Simulations of AUC_0–24h_ in the typical patient of the HIGHRIF1 study population at steady state ranged from 122.2 h. mg/L to 331.2 h. mg/L (95% PI), i.e. the rifampicin exposure varied by 270% between occasions solely due to IOV. This translates to a tripling of the dose from one occasion to another, assuming linear PK. Rifampicin however exhibits a more than dose-proportional increase in exposure, which is due to its dose-dependent bioavailability and concentration-dependent clearance (Svensson et al., 2018a). At higher doses, efflux transporters and/or enzymes in the gut wall become saturated, which leads to an increase in bioavailability. In addition, biliary excretion and transporters in the liver become saturated with increasing rifampicin plasma concentrations, and thus clearance decreases. Those phenomena lead to a nonlinear increase in exposure with higher doses, i.e. higher increase in exposure with increased dose compared to what is expected for linear PK (Acocella et al., 1972; Svensson et al., 2018a). When taking this non-linearity into account, as it has been done in the POPPK model used in this work (Svensson et al., 2018a), the 270% difference in AUC_0–24h_ translates to almost a 200% difference on a dose level. This means that the exposure (AUC_0–24h_) within the same individual varies at different days, or sampling occasions, even if the patient is in steady state. Therefore, it is difficult to forecast the exposure, and subsequently the dose at the next occasion, as illustrated in **Figure 3**. In this situation, it would be challenging to use a traditional approach such as TDM, since the decision on decreasing, increasing, or continuing the current dose is based on the observed exposure in relation to a target. Since IOV occurs at random, the decision to decrease, increase or continue the dose would be highly influenced by the IOV, which would result in different decisions being made at every occasion, which subsequently would lead to a high fluctuation in dose recommendations. This is the reason why it is generally believed that TDM is of low value when a drug has high IOV compared to IIV (Karlsson and Sheiner, 1993; Wallin et al., 2010; Holford and Buclin, 2012; Liefaard and Chen, 2015). However, it has recently been demonstrated that if IOV is taken into account properly, i.e. in a MIPD approach, dose individualization is also possible for drugs with high IOV, predicting one dose for all dosing occasions (Wicha and Hennig, 2018; Abrantes et al., 2019). In this MIPD approach, IOV is included in the POPPK model to estimate EBEs accurately, but disregarded in the prediction of AUC_0–24h_ which is used for the subsequent dose predictions. Ignoring IOV in the prediction of the AUC_0–24h_ at the next occasion, results in reduction of noise in the dose predictions. In this respect, it should be underlined that the acceptable range of AUC_0–24h_ is a Bayesian range which was derived excluding IIV and IOV, i.e. the acceptable range of AUC_0–24h_ cannot be compared to an observed AUC_0–24h_ range as the Bayesian range is a narrower AUC_0–24h_ range, compared to what is observed after a dose. This is why a Bayesian acceptable range of AUC_0–24h_ cannot be applied directly to observed AUC_0–24h_ values.

In order to evaluate the need to account for IOV in the underlying model when performing dose individualization of rifampicin, we re-estimated the original POPPK model (Svensson et al., 2018a) by fixing IOV in all parameters to zero, which led to deviations in both fixed and random effects parameters (**Table 4**). Ignoring IOV in the estimation when there is in truth IOV present, resulted in an underestimation of clearance, overestimation of volume of distribution, and an increase in IIV and residual error (**Table 4**), which is in accordance with the findings by Karlsson and Sheiner (1993). It has been shown that ignoring IOV will increase IIV, because IOV gets lumped together with either IIV or residual error. To avoid describing some IIV mistakenly as IOV, the IIV should be included in the model first, and the remaining variability explained by IOV (Karlsson and Sheiner, 1993). In our simulations, the IIV in k_a_ was lower in the re-estimated model without IOV, compared to in the original model including IOV, which was unexpected. However, this parameter was estimated with high uncertainty (**Table 4**). More importantly, it could be demonstrated in this work, that if IOV was ignored in the estimation of EBEs for MIPD performance, the individual prediction error increased compared to when IOV was accounted for in the underlying model (**Figure 6**). We hypothesize further that the individual prediction error in predicted doses using a MIPD approach is lower compared to when traditional TDM is performed, since an MIPD approach suggests a specific dose leading to the desired exposure, while in TDM the clinician only receives information on if the dose should be increased, decreased, or kept. This has to be confirmed in further simulation studies.

Individualized dosing is crucial to ensure optimal rifampicin exposure, since treatment failure, resistance development, and relapse of TB disease have been linked to suboptimal rifampicin plasma concentrations (Pasipanodya et al., 2013; Alsultan and Peloquin, 2014; Ramachandran et al., 2019). To prevent prolonged treatment, which increases the patient’s risk for adverse events and treatment costs, it is critical to use approaches ensuring that adequate drug exposure is achieved in every patient, which can be accomplished through MIPD. The moderate variability in AUC_0–24h_ between patients identified in this study (25.4%) can be reduced through MIPD in order to increase the efficacy. However, although the variability in exposure between individuals is reduced with MIPD, as shown in **Figure 7**, the range of true individual doses needed for each individual patient to reach an exposure within the same acceptable range of AUC_0–24h_ at day 14 (189–224 h. mg/L) is very wide, ranging from 600 to 2700 mg. With MIPD it is possible to perform dose individualization at any time, even before steady state has been reached, and to keep the number of plasma samples to a minimum. However, since rifampicin PK properties are very complex, not all approaches are appropriate for dose individualization. The method has to handle rifampicin auto-induction, dose-dependent bioavailability, concentration-dependent clearance, and high IOV in order to predict individual doses correctly (Svensson et al., 2019), which is achieved with our MIPD approach.

This work and evaluation is based on a fixed sampling design for the PK samples, i.e. samples taken pre-dose and at 2 and 4 h post-dose. As EBEs and shrinkage are dependent on the sampling design, different EBEs for each individual would have been achieved with a different sampling design. However, the sampling PK design used in this work is supported by the work by van Beek et al. (2019) who identified this sampling design as most informative for deriving EBEs of rifampicin among those evaluated. If a different design for any reason would be applied, the absolute dose prediction error would most likely be higher. However, the difference in individual prediction error between the approach where IOV is included in the EBE estimation and the approach where IOV is ignored, would most likely remain the same. Furthermore, the MIPD approach utilized in this work assumes that a time-varying target is needed in order to empirically mimic the time varying exposure in patients caused by auto-induction of rifampicin elimination.

The MIPD approach used in this simulation study including IOV in the EBE estimation, where information from two sampling occasions (days 1 and 7) with three samples per occasion (pre-dose, 2 and 4 h post-dose) were incorporated, performed well. The median of the individual prediction error was 0%, and the 75% PI in individual prediction error ranged from −14.6% to 0.0% and the 95% PI from −20.0% to 25.0%, respectively (**Figure 6**). In the cases where the dose was not predicted correctly, it was more often underpredicted, that is a prediction of a lower dose compared to the true dose, which is due to the design of the dosing algorithm, that will always choose the lowest dose possible, as described in *Model-Informed Precision Dosing Algorithm*. This results in a negatively biased and left-skewed individual prediction error which is favorable from a safety point of view. The current standard of care dose for rifampicin is 10 mg/kg. Based on recent studies as described in our paper, doses up to 35 mg/kg have been proven on average to be safe. This is however only based on short term data in a limited number of patients this far. As safety data still is limited for this 3.5-fold higher target than standard of care, an algorithm that predicts toward the lower end of the acceptable AUC_0–24h_ interval is more safe. As more clinical data and reports becomes available over time which supports that 35 mg/kg is a very safe target, the individualized dosing target could be changed and not favor the lower end of the acceptable AUC_0–24h_ interval. Most importantly, the mode of the true dose achieving the target exposure, which was 1,500 mg in the population, was forecasted correctly in 95% of the cases (**Figure 7**). Even though the Bayesian acceptable range of AUC_0–24h_ was derived around 35 mg/kg, which translates to 1,800 mg in the typical patient (WT: 53.9 kg), the mode of the true optimal individualized dose in the simulated study population was 1,500 mg (30 mg/kg). This was due to the WT distribution in the HIGHRIF1 study population, which was used to create the covariate distribution in this simulation study. The mode in WT was lower in this simulation study population (52.4 kg) than the median WT (53.9 kg), and therefore more simulated patients received an individualized dose of 1,500 mg (n = 325) to reach the target compared to simulated patients receiving 1,800 mg (n = 292) (**Figure 7**). This shows that depending on the study population, the distribution of required doses to reach the target can vary, i.e. even if the target exposure is set to the exposure in a typical patient after a dose of 35 mg/kg in the HIGHRIF1 trial, the most common dose (mode) will not be 35 mg/kg in every population. This can be due differences in covariates (e.g. WT, FFM, or clearance) in the population compared to the typical individual of the HIGHRIF1 study population.

Besides high accuracy in dose predictions, another advantage of a MIPD approach is the limited amount of samples and sampling occasions needed to forecast the next dose. It has previously been shown, that taking merely two blood samples (2 and 4 h post-dose) is sufficient to characterize individual PK parameters (van Beek et al., 2019) when using a model-based approach. In addition, the results of this simulation study demonstrate that two sampling occasions are sufficient to capture the IOV and individual exposure, since the decrease in imprecision and bias in dose predictions was statistically significant when information from two occasions were used to estimate EBEs, compared to only using information from a single occasion (**Tables 5** and **6**). However, when adding information from a third occasion, improvement in precision and accuracy was not statistically significant (**Tables 5** and **6**). Thus, when using a MIPD approach, it is sufficient to take PK samples at day 1 after start of treatment and at one additional sampling occasion during the following week to determine the EBEs of the individual PK parameters. Sampling can be done at additional occasions when drug-drug interactions are suspected, where lower or higher rifampicin plasma concentrations are anticipated and where dose adjustment is needed. This could be investigated by taking additional plasma samples and derive an optimized dose using the MIPD approach. This should be seen as a new situation, and a new set of two sampling occasions with the same set up, i.e. a pre-dose sample and a 2 and 4 h post-dose sample taken one week apart, should be performed.

In conclusion, our work demonstrates that MIPD can be a valuable tool for individualized dosing of drugs with high IOV in exposure and that IOV should be accounted for in the estimation of EBEs but excluded when forecasting the most optimal dose, as demonstrated here with rifampicin as an example. A large variability in exposure of rifampicin between occasions was shown in this work. In order to forecast the next individual dose correctly, IOV must be acknowledged which can be achieved using a MIPD approach with PK information from at least two sampling occasions.

## Data Availability Statement

The raw data supporting the conclusions of this article will be made available by the authors, without undue reservation, to any qualified researcher.

## Ethics Statement

No ethics approval of an ethics committee and written informed consent from participants had to be obtained, since all data was simulated and thus no personal data was handled.

## Author Contributions

LK and US contributed equally to this work. LK and US carried out the simulations, interpreted the results, wrote and edited the manuscript. LK and US read and approved the submitted version.

## Conflict of Interest

The authors declare that the research was conducted in the absence of any commercial or financial relationships that could be construed as a potential conflict of interest.
