# Supplementary material for: Individualized Dosing With High Inter-Occasion Variability Is Correctly Handled With Model-Informed Precision Dosing—Using Rifampicin as an Example
Source: Front Pharmacol. 2020 May 27;11:794. doi: 10.3389/fphar.2020.00794 (PMC7266983; doi:10.3389/fphar.2020.00794)
Supplement: Supplementary file 1 [file DataSheet_1.docx]

**Supplementary appendix S1 Final NM-TRAN model code for re-estimated model without IOV**

$PROBLEM POPPK model re-estimated without IOV

$ABBREVIATED DERIV2=NOCOMMON

$INPUT ID TIME TADO DGRP DV BQL AMT EVID OCC PLOT AGE SEX RACE WT HT BMI HIV FFM NDV DOSE

; TIME=hours, TADO=time after last dose, DGRP=dose in mg/kg, BQL (0=observation is not BLOQ, 1=observation is BLOQ, 2=observation missing, 3=dummy or dosing time point), OCC (1=day 1, 2=day 7, 3=day 14), PLOT=flag variable for creating VPCs, SEX (1=male, 0=female), FFM=fat free mass in kg, DOSE=dose in mg

$DATA ... IGNORE=@

$SUBROUTINE ADVAN13 TRANS1 TOL=10

$MODEL NCOMP=3 COMP=(DEPOT,DEFDOSE) COMP=(CENTRAL,DEFOBS)

COMP=(ENZ)

$PK

"FIRST

" COMMON/PRCOMG/ IDUM1,IDUM2,IMAX,IDUM4,IDUM5

" INTEGER IDUM1,IDUM2,IMAX,IDUM4,IDUM5

" IMAX=1000000000

IF(AMT.GT.0)PD = AMT ; PD = oral DOSE

IF(AMT.GT.0)TDOS = TIME ; TDOS = time of DOSE

TAD = TIME - TDOS ; TAD = time after DOSE

;;--- ----- Allometric scaling using fat-free mass ----------

NFMCL = FFM

ALLMCL = (NFMCL/70)**0.75

NFMV = FFM

ALLMV = (NFMV/70)

;----Typical parameters--------------------------------------

TVVMAX = THETA(1) ; VMAX (MG/H/70KG)
TVKM = THETA(2) ; KM (MG/L)
TVV2 = THETA(3)*ALLMV ; V2 (L/70KG)
TVKA = THETA(4) ; KA (H-1)
TVEMAX = THETA(5) ; EMAX
TVEC50 = THETA(6) ; EC50 (MG/L)
TVKENZ = THETA(7) ; KENZ (H-1)
TVMTT = THETA(8) ; MTT (H)
TVNN = THETA(9) ; NN
TVFEMAX = THETA(10) ; FEMAX
TVFED50 = THETA(11) ; ED50 (MG)

;----Inter-occasion variability------------------------------

IF (OCC.EQ.1) THEN

IOVBIO = ETA(7)

ELSE

IOVBIO = ETA(8)

ENDIF

IF (OCC.EQ.1) THEN

IOVMTT = ETA(9)

ELSE

IOVMTT = ETA(10)

ENDIF

IF (OCC.EQ.1) THEN

IOVKM = ETA(11)

ELSE

IOVKM = ETA(12)

ENDIF

IF (OCC.EQ.1) THEN

IOVKA = ETA(13)

ELSE

IOVKA = ETA(14)

ENDIF

;----Individual parameters-----------------------------------

VMAX = TVVMAX*EXP(ETA(2))

KM = TVKM*EXP(ETA(1)+IOVKM)

V2 = TVV2*EXP(ETA(3))

KA = TVKA*EXP(ETA(6)+IOVKA)

EC50 = TVEC50

EMAX = TVEMAX

KENZ = TVKENZ

FEMAX = TVFEMAX

FED50 = TVFED50

TVBIO = 1*(1+FEMAX*(DOSE-450)/(FED50+(DOSE-450)))

BIO = TVBIO*EXP(IOVBIO)

K = CL/V2

MTT = TVMTT*EXP(ETA(4)+IOVMTT)

NN = TVNN*EXP(ETA(5))

S2 = V2

;----Initialization of compartments---------------------

F1 = 0 ; Transit absorption compartment

A_0(2) = 0.0001 ; Central compartment

A_0(3) = 1 ; Induction compartment

KTR = (NN + 1) / MTT

;----Logarithm of the approximation to the gamma function

L = 0.9189385 + (NN + 0.5)*LOG(NN) - NN + LOG(1 + 1/(12*NN))

LBPD = LOG(BIO*PD)

LKTR = LOG(KTR)

CUMUL = LBPD + LKTR - L

$DES
CP = A(2)/V2 ; Rifampicin plasma concentration (mg/L)

TEMPO = T – TDOS
 IF(TEMPO.GT.0)THEN
 KTT = KTR*TEMPO
 DADT(1) = EXP(CUMUL + NN*LOG(KTT) - KTT) - KA*A(1)
 ELSE
 KTT = 0
 DADT(1) = 0
 ENDIF

DADT(2) = KA*A(1) - (((VMAX/(KM+CP))*ALLMCL)/V2)*A(2)*A(3)
EFF = (EMAX*(CP)) / (EC50 + CP)
DADT(3) = KENZ*(1 + EFF) - KENZ*A(3)

$ERROR
IPRED = LOG(A(2)/S2+0.00001) ; concentration in central compartment (log transformed)
ADD = SQRT(SIGMA(1,1)) ; additive error with log DV (approximates proportional error on normal scale)
SD = SQRT((ADD)**2)

;Sim_start
LLOQ=LOG(0.13)

DUM=(LLOQ-IPRED)/SD
CUMD=PHI(DUM)

IF(DV.GE.LLOQ) THEN
F_FLAG = 0
IRES = DV – IPRED
IWRES = IRES / SD
Y = IPRED + EPS(1)
ELSE
F_FLAG = 1
IRES = 0
IWRES = 0
MDVRES = 1
Y=CUMD
ENDIF

;IRES = DV – IPRED
;IWRES = IRES / SD
;Y = IPRED + EPS(1)
;Sim_end

;----Output--------------------------------------
AA1 = A(1) ; ABSORPTION COMPARTMENT
AA2 = A(2) ; CENTRAL RIFAMPICIN COMPARTMENT
AA3 = A(3) ; INDUCTION COMPARTMENT

IF(AMT.GT.0) THEN
 TDOS = TIME
 PD = AMT
ENDIF

$THETA (0,310) ; 1 VMAX

$THETA (0,15.8) ; 2 KM

$THETA (0,93.6) ; 3 V2

$THETA (0,2.4) ; 4 KA

$THETA (0,1.219,1.25) ; 5 EMAX

$THETA (0,0.05256) ; 6 EC50

$THETA (0.005,0.005328) ; 7 KENZ

$THETA (0,0.812) ; 8 MTT

$THETA (1,7.568) ; 9 NN

$THETA (0,0.401) ; 10 FEMAX

$THETA (0,17.35) ; 11 FED50

$OMEGA BLOCK(2)

0.8062 ; 1 IIV in KM

0.5957 0.5524 ; 2 IIV IN VMAX

$OMEGA 0.08615 ; 3 IIV in V2

$OMEGA 0.5875 ; 4 IIV in MTT

$OMEGA 0.9048 ; 5 IIV in NN

$OMEGA 0.2868 ; 6 IIV in KA

$OMEGA BLOCK(1) 0 FIX ; 7 IOV in F

$OMEGA BLOCK(1) SAME

$OMEGA BLOCK(1) 0 FIX ; 9 IOV in MTT

$OMEGA BLOCK(1) SAME

$OMEGA BLOCK(1) 0 FIX ; 11 IOV in KM

$OMEGA BLOCK(1) SAME

$OMEGA BLOCK(1) 0 FIX ; 13 IOV in KA

$OMEGA BLOCK(1) SAME

$SIGMA 0.57 ; ADD ERROR

;Sim_start

$ESTIMATION METHOD=1 LAPLACIAN INTER NUMERICAL SLOW MAXEVAL=9999 NSIG=3 SIGL=9 PRINT=3 MCETA=100 NOABORT

$COVARIANCE PRINT=E MATRIX=S SLOW

;$SIMULATION (1234) ONLYSIM

;Sim_end

$TABLE ID IPRED IWRES CWRES NPDE DV OCC TIME TADO DGRP PLOT CP NOPRINT ONEHEADER FILE=sdtab

$TABLE ID NOPRINT ONEHEADER FILE=catab

$TABLE ID AGE SEX RACE WT HT BMI HIV FFM NOPRINT ONEHEADER FILE=cotab

$TABLE ID V2 MTT BIO NN KM KA VMAX ETA1 ETA2 ETA3 ETA4 ETA5 ETA6 ETA7 ETA8 ETA9 ETA10 ETA11 ETA12 ETA13 NOPRINT ONEHEADER FILE=patab

$TABLE ID NOPRINT ONEHEADER FILE=mytab

**Supplementary appendix S2 R code for calculation of individualized dose**

# Script to determine the individualized dose

# Always clean workspace

rm(list=ls())

# Change work path as needed

setwd("/..")

library(tidyverse)

require(ggplot2)

require(xpose4)

library(gridExtra)

aucfunc <- function(x, # the id

dfname=1, # The dataframe name

lowlim=0, # lower limit

upplim=10, # upper limit

day=1 # day

) {

# Read in data

dat <- eval(parse(text=dfname)) %>% filter(ID==x & TIME==day)

# Find out if any AUC falls within limit

if(nrow(dat %>% filter(AUC>lowlim & AUC<upplim))==1) {

subdat <- dat %>% filter(AUC>lowlim & AUC<upplim)

REQDOSE <- subdat$DOSE

REQDGRP <- subdat$DGRP

AUC <- subdat$AUC

}

# If more than 1 AUC falls within limit then choose the

# value closest to the lower limit

if(nrow(dat %>% filter(AUC>lowlim & AUC<upplim))>1) {

# Subset to include all values within range

subdat <- dat %>% filter(AUC>lowlim & AUC<upplim) %>%

# Filter to only include the dose/AUC for the AUC closest to the lower limit

mutate(DIFF=case_when(

AUC<=mean(c(lowlim,upplim)) ~ AUC-lowlim,

AUC>mean(c(lowlim,upplim)) ~ AUC-lowlim,

TRUE ~-99

)) %>%

# Filter to only include the row with the lowest DIFF

filter(DIFF==min(DIFF))

REQDOSE <- subdat$DOSE

REQDGRP <- subdat$DGRP

AUC <- subdat$AUC

}

# If no AUC falls within limit then choose the

# value closest to the midpoint of the limit

if(nrow(dat %>% filter(AUC>lowlim & AUC<upplim))==0) {

subdat <- rbind(

# Find highest AUC within the range

dat %>% filter(AUC<lowlim) %>% filter(AUC==max(AUC)),

# Find the lowest AUC within the range

dat %>% filter(AUC>upplim) %>% filter(AUC==min(AUC))

) %>%

# Filter to only include the dose/AUC for the AUC closest to the

# upper and/or lower limit

mutate(DIFF=case_when(

AUC==min(AUC) ~ lowlim-AUC,

AUC==max(AUC) ~ AUC-upplim,

TRUE ~-99

)) %>%

# Filter to only include the row with the lowest DIFF

filter(DIFF==min(DIFF))

REQDOSE <- subdat$DOSE

REQDGRP <- subdat$DGRP

AUC <- subdat$AUC

}

return(c("ID"=x,"DOSE"=REQDOSE,"DGRP"=REQDGRP,"AUC"=AUC))

}

# Add/read in the data

# Read in the individual AUC and dose predictions

AUCdata <- read.csv("Predictions_rifampicin_all_doses_sdtab1.csv")

# Subset the predictions dataframe as relevant

comb <- AUCdata %>%

# Make a DGRP column. mutate(DGRP=rep(seq(from=10,to=55,by=5),each=1000,times=3)) %>%

# Filter out days 1 and 28

#filter(TIME!=1) %>% filter(TIME!=28) %>%

mutate(PDAY=ifelse(TIME==14,"Day 14","Steady state")) %>%

# Limit the doses to 50 mg/kg

filter(DGRP<=50)

# Read in all target values

all_targets <- read.csv("all_targets.csv")

AUC_target_d1 <- c(all_targets[4,]$lower,all_targets[4,]$upper)

AUC_target_d7 <- c(all_targets[5,]$lower,all_targets[5,]$upper)

AUC_target_d14 <- c(all_targets[6,]$lower,all_targets[6,]$upper)

# Calculate dose distribution according to AUC-based range

raw_results_range_AUC <- unlist(lapply(unique(comb$ID),aucfunc,dfname="comb",

day=1,lowlim=AUC_target_d1[1],

upplim=AUC_target_d1[2]))

# Reformat results into df

results_range_AUC <- tibble(

ID=raw_results_range_AUC[seq(from=1,to=length(raw_results_range_AUC),by=4)],

REQDOSE=raw_results_range_AUC[seq(from=2,to=length(raw_results_range_AUC),by=4)],

REQDGRP=raw_results_range_AUC[seq(from=3,to=length(raw_results_range_AUC),by=4)],

AUC=raw_results_range_AUC[seq(from=4,to=length(raw_results_range_AUC),by=4)]

) %>% # Calculate the % that is outside the target

mutate(outside=ifelse(AUC<AUC_target_d1[2] & AUC>AUC_target_d1[1],0,1)) %>%

mutate(perc_outside=case_when(

outside==1 & AUC<AUC_target_d1[1] ~ (AUC_target_d1[1]-AUC)/(AUC_target_d1[1])*100,

outside==1 & AUC>AUC_target_d1[2] ~ (AUC_target_d1[2]-AUC)/(AUC_target_d1[2])*100,

TRUE ~ -99

))

# Calculate table w % patients per dose

perc_reqdose_AUC <- results_range_AUC %>% group_by(REQDGRP) %>% summarize(n_DGRP=n()/24*100)

write.csv(results_range_AUC, file="output_day1.csv")
